# Aluminum Responsive Genes in Flax (*Linum usitatissimum* L.)

**DOI:** 10.1155/2019/5023125

**Published:** 2019-02-28

**Authors:** George S. Krasnov, Alexey A. Dmitriev, Alexander V. Zyablitsin, Tatiana A. Rozhmina, Alexander A. Zhuchenko, Parfait Kezimana, Anastasiya V. Snezhkina, Maria S. Fedorova, Roman O. Novakovskiy, Elena N. Pushkova, Liubov V. Povkhova, Nadezhda L. Bolsheva, Anna V. Kudryavtseva, Nataliya V. Melnikova

**Affiliations:** ^1^Engelhardt Institute of Molecular Biology, Russian Academy of Sciences, Moscow 119991, Russia; ^2^Federal Research Center for Bast Fiber Crops, Torzhok 172002, Russia; ^3^All-Russian Horticultural Institute for Breeding, Agrotechnology and Nursery, Moscow 115598, Russia; ^4^Peoples' Friendship University of Russia (RUDN University), Moscow 117198, Russia; ^5^Moscow Institute of Physics and Technology, Dolgoprudny 141701, Russia

## Abstract

Flax (*Linum usitatissimum* L.) is a multipurpose crop which is used for the production of textile, oils, composite materials, pharmaceuticals,* etc*. Soil acidity results in a loss of seed and fiber production of flax, and aluminum toxicity is a major factor that depresses plant growth and development in acid conditions. In the present work, we evaluated gene expression alterations in four flax genotypes with diverse tolerance to aluminum exposure. Using RNA-Seq approach, we revealed genes that are differentially expressed under aluminum stress in resistant (Hermes, TMP1919) and sensitive (Lira, Orshanskiy) cultivars and selectively confirmed the identified alterations using qPCR. To search for differences in response to aluminum between resistant and sensitive genotypes, we developed the scoring that allowed us to suggest the involvement of MADS-box and NAC transcription factors regulating plant growth and development and enzymes participating in cell wall modifications in aluminum tolerance in flax. Using Gene Ontology (GO) enrichment analysis, we revealed that glutathione metabolism, oxidoreductase, and transmembrane transporter activities are the most affected by the studied stress in flax. Thus, we identified genes that are involved in aluminum response in resistant and sensitive genotypes and suggested genes that contribute to flax tolerance to the aluminum stress.

## 1. Introduction

Among abiotic stresses, aluminum (Al) toxicity is a major constraint for crop production in acid soils worldwide [1]. In acidic conditions, the mineral form of Al dissolves to release the soluble Al^3+^ form, which is capable of crossing the plant membranes and is highly toxic to plants that even micro concentrations can inhibit root growth within minutes or hours in many agricultural plant species [2–5]. Al negatively affects cell elongation and division, uptake and transport of nutrients, and Ca^2+^ homeostasis and disturbs the structure and function of the plasma membrane, cell wall, and chromatin [6–10].

The mechanisms of resistance to Al are diverse in plants and could be divided into exclusion, which decreases the amount of phytotoxic Al^3+^ in the cells and internal tolerance, which reduces Al toxicity in root and shoot symplast [6, 11–19]. Excretion of Al detoxifying organic acids (OAs) to the apoplast or rhizosphere is the most common variant of exclusion, in which genes encoding the OA transporters, including aluminum-activated malate transporter 1 (ALMT1) and members of the multidrug and toxic compound extrusion (MATE) citrate transporter gene family, are involved [13, 20]. Exudation of mucilage also binds Al ions and results in the exclusion of Al and its detoxification [21–23]. Mechanism of Al tolerance encompasses processes that result in chelation of cytosolic Al^3+^ with organic ligands, sequestration into the vacuole, or transport of Al^3+^ to less-sensitive regions of the plant [24–26]. Recovery from damages following exposure to Al toxicity is mediated by the detoxification of the reactive oxygen species (ROS) through ROS-detoxifying enzymes, such as glutathione S-transferases, peroxidases, and superoxide dismutases that confer Al tolerance [20, 27, 28]. Significant genetic diversity in Al resistance or tolerance was found in crops that enable development of improved cultivars, which maintain yield on acid soil [29, 30].

For flax (*Linum usitatissimum *L.), a crop grown worldwide for fiber and seeds and also known for its health benefits [31, 32], the mechanisms for Al tolerance are little known, although Al toxicity in acid soils is a serious problem for cultivation and rich harvest of flax [33]. Therefore, it is imperative to understand the molecular mechanisms underlying Al tolerance in flax. High-throughput sequencing methods are extensively used for evaluation of expression alterations of protein-coding genes or miRNAs in flax under diverse stresses [34–41]. We have previously shown that UDP-glycosyltransferases, glutathione S-transferases, and Ca^2+^/H^+^-exchanger CAX3 are involved in flax tolerance to aluminum [42, 43]. miR319, miR390, and miR393 also participate in aluminum response* via* regulation of growth processes [44]. However, Al tolerance mechanisms are complex and could involve diverse strategies. In the present work, we conducted comprehensive gene expression analysis of flax cultivars grown under control and Al-treated conditions to reveal genes that participate in Al response and to identify differences in gene expression alterations between resistant and sensitive to aluminum genotypes.

## 2. Materials and Methods

### 2.1. Plant Material

A laboratory method for evaluation of flax tolerance to aluminum, whose results have high correlation with field assessment, was taken as the basis for creating aluminum treatment conditions [45]. Resistant and sensitive cultivars for the study were chosen on the basis of our field and laboratory experiments, in which productivity indexes and root length were used for assessment of flax genotype tolerance to soil acidity and aluminum [33]. For the present study of gene expression alterations under short-term (4 h) aluminum exposure (500 *μ*M AlCl_3_) at low pH (4.5), resistant (Hermes and TMP1919) and sensitive (Lira and Orshanskiy) to aluminum stress flax cultivars were used. Flax seeds were germinated in Petri dishes on filter paper and then were transferred into 50 mL Falcon tubes with filter paper soaked in 0.5 mM CaCl_2_ pH 4.5 for 24 h. Seedlings in control conditions were then grown for 24 h more, while seedlings in stress conditions were grown for 20 h followed by addition of 500 *μ*M AlCl_3_ for 4 h. After that, root tips, 8-10 mm in length, were collected and immediately frozen in liquid nitrogen. The samples were stored at -70°С. RNA was extracted using RNA MiniPrep kit (Zymo Research, USA) and then used for transcriptome sequencing on HiSeq2500 (Illumina, USA) with paired-end 100-nucleotide reads in two biological replicates as described in our previous work [42].

### 2.2. Identification of Differentially Expressed Genes

Transcriptome assembly and annotation were performed using Trinity and Trinotate as described earlier [42]. The reads of each cultivar (Hermes, TMP1919, Lira, and Orshanskiy) were mapped to the assembled transcripts of Hermes using bowtie2 [46] and quantified using RSEM [47]. Then, read count data were analyzed using edgeR package [48]. Genes with read counts per million (CPM) below 1.5 were filtered out. Normalization using the TMM method was performed and genes with expression alterations under aluminum treatment were identified within the following groups:

1. pool of all analyzed flax genotypes;
2. pool of resistant cultivars (Hermes and TMP1919);
3. pool of sensitive cultivars (Lira and Orshanskiy);
4. individual genotypes (Hermes, TMP1919, Lira, or Orshanskiy).

To evaluate expression alterations for each transcript, expression level fold change (*FC*) was calculated for each gene as the ratio of CPM under Al^3+^ exposure to CPM under control conditions.

To identify genes with diverse expression alterations in resistant and sensitive to aluminum genotypes, we calculated delta-score (*S*_Δ_) that takes into account (1) consistency of gene expression changes within each group (resistant or sensitive cultivars), (2) magnitude of these changes, and (3) differences between groups. To proceed this way, we derived consistency scores for resistant and sensitive cultivars, which correspond to the first component:

(1) $$\begin{array}{c} C_{res.}=\frac{{\left(\sum_{res.}\sqrt[{3}]{\log{FC}_{i}}\right)}^{3}}{\max\left(\left|\log{FC}_{i}\right|\right)} \end{array}$$

where log⁡*FC* is the binary logarithm of* FC*, and i = 1, 2 (number of resistant genotypes). Hence, if aluminum-induced gene expression changes are unidirectional and* FC* values are close to each other, *C*_res._ would have the greatest value. In the similar way, we calculated the consistency score for sensitive cultivars (*C*_sens._). Finally, delta-scores (*S*_Δ_) were calculated as

(2) SΔ=Cres.×Csens.×maxres.,sens. ⁡meanlog⁡FCi×meanres.⁡log⁡FCi−meansens.⁡log⁡FCi2

Here, the 1^st^ multiplier reflects the consistency of gene expression changes, the 2^nd^ one—their maximum magnitude, and the 3^rd^ one—differences between groups. We introduced 2-fold greater weight for the 3^rd^ component because of its prime importance.

### 2.3. Quantitative PCR (qPCR) Analysis

For validation of high-throughput sequencing gene expression data, qPCR was used. The same RNA samples of Hermes, TMP1919, Lira, and Orshanskiy that were used for high-throughput sequencing were analyzed. RevertAid Reverse Transcriptase (Thermo Fisher Scientific, USA) was used to generate first-strand cDNA. Transcripts for qPCR analysis were chosen on the basis of transcriptome sequencing data. The selected transcripts encode the following proteins: LHY (TR32133∣c1_g1), transcription factor (TF) LUX (TR44570∣c0_g2), putative lysine-specific demethylase JMJ30 (TR19190∣c0_g1), high mobility group B protein 1 (TR12175∣c1_g1), zinc finger protein CONSTANS-like 10 (TR53996∣c0_g1), high-affinity nitrate transporter 3.1 (TR26619∣c0_g1), and two-component response regulator-like APRR1 (TR32104∣c0_g2). Primers and probes were designed using ProbeFinder Software (Roche, Switzerland) (Table 1). qPCR was performed on 7500 Real-Time PCR System (Applied Biosystems, USA). Reaction mix contained 1x PCR buffer (GenLab, Russia), 250 nM of dNTPs (Fermentas, Lithuania), 300 nM of each primer, 200 nM of short hydrolysis probes from Universal ProbeLibrary (Roche), 1 U of Taq polymerase (GenLab), and cDNA. The following qPCR program was used: 95°C for 10 min, 50 cycles of 95°C for 15 s, and 60°C for 60 s.* ETIF3E* was used as a reference gene [35, 49]. All reactions were performed in three technical replicates. All the calculations were carried out using our ATG tool [35, 50]. ΔΔ*C*_*t*_ values were calculated for the assessment of gene expression level [35, 37]. Correlation between qPCR (ΔΔ*C*_*t*_) and high-throughput sequencing (log⁡*FC*) data was evaluated by Spearman's correlation coefficient.

### 2.4. Gene Ontology Analysis

The gene set enrichment analysis (GSEA) based on Gene Ontology (GO) data was performed using Goseq package (http://bioconductor.org/packages/release/bioc/html/goseq.html). Analysis of top lists of up- and downregulated genes was carried out for the pool of all analyzed genotypes, the pool of resistant cultivars, the pool of sensitive cultivars, and individual genotypes for identification of enriched GO terms. Heatmaps illustrating gene expression alterations in selected GO terms were created using R package pheatmap. Weighted gene correlation network analysis was carried out using R package WGCNA [51] for genes with CPM > 20 in at least 6 samples.

## 3. Results and Discussion

### 3.1. The Effects of Aluminum Exposure at Low pH on Flax Plants

Soil acidity and Al^3+^ ions result in depression of flax plants, inhibition of growth, changes in height, fiber mass, and seed productivity both in resistant and sensitive cultivars. However, the degree of changes varied between genotypes [33]. For the present study, we used flax cultivars with contrast extent of phenotypic changes in acid soils with high aluminum content (pH 4.0, Al content – 11.07 mg/100 g of soil). Results of field experiments for Hermes, ТМР1919, Lira, and Orshanskiy cultivars under control conditions and low pH with Al treatment are presented in Table 2. Cultivars Hermes and ТМР1919 showed greater tolerance compared to Lira and Orshanskiy that was especially noticeable for fiber mass and a number of seed pods.

In laboratory experiments, inhibition of root growth was observed in flax plants under aluminum exposure at low pH (pH 4.5, 500 *μ*M AlCl_3_). Negative effects were more pronounced in sensitive cultivars, where 50-60% reduction in root length was revealed after 5 days, while in resistant cultivars reduction was less than 30% [45].

### 3.2. Genes with Differential Expression under Al^3+^ Exposure

As shown in our previous works, even after 4 h of Al^3+^ exposure when phenotype changes were not yet noticeable, significant gene expression alterations occurred in flax plants [42, 44]. These results are consistent with the studies on other plant species, for many of which short response time to aluminum was revealed [19]. Therefore, in the present work, we focused on the effects of short-term aluminum treatment and evaluated gene expression alterations after 4 h of aluminum exposure in resistant and sensitive to the studied stress flax genotypes based on transcriptome sequencing data. In this regard, in contrast to our previous study [42], we compared gene expression levels in flax plants under control conditions and after 4 h of Al^3+^ exposure in the pool of all studied genotypes, groups of resistant and sensitive plants, or individual cultivars. Results of our analysis are presented in Supplementary Materials for the pool of all studied genotypes (S1 table), pool of resistant (Hermes and TMP1919, S2 Table) and pool of sensitive (Lira and Orshanskiy, S3 Table) genotypes, and individual cultivars (S4, S5, S6, and S7 Tables for Hermes, TMP1919, Lira, and Orshanskiy respectively), where the genes are listed in the order of decreasing statistical significance of expression alterations.

**Figure 1:**
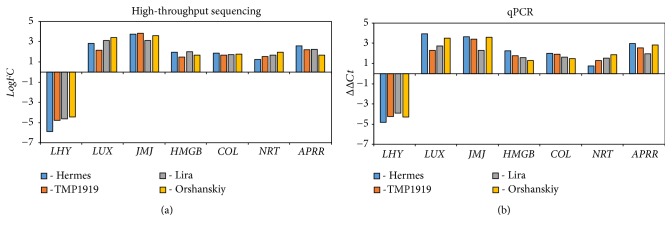
Expression alterations of 7 genes in flax cultivars Hermes, TMP1919, Lira, and Orshanskiy under aluminum exposure at low pH evaluated by high-throughput sequencing (a) and qPCR (b).

To validate high-throughput sequencing data, we evaluated mRNA level of transcripts with significant expression alterations under the studied stress in flax cultivars grown under control conditions and Al^3+^ exposure using qPCR. Expression of genes encoding protein LHY (TR32133∣c1_g1), TF LUX (TR44570∣c0_g2), putative lysine-specific demethylase JMJ30 (TR19190∣c0_g1), high mobility group B protein 1 (TR12175∣c1_g1), zinc finger protein CONSTANS-like 10 (TR53996∣c0_g1), high-affinity nitrate transporter 3.1 (TR26619∣c0_g1), and two-component response regulator-like APRR1 (TR32104∣c0_g2) was analyzed. Reaction efficiencies were 95% or higher, and Ct (cycle threshold) values varied from 23 to 32. As seen from Figure 1, the expression data obtained by high-throughput sequencing (Figure 1(a)) and qPCR (Figure 1(b)) were highly consistent that indicates the reliability of the sequencing data. Spearman's correlation coefficient was 0.89 (*p* < 0.01).

We performed the search for differences in transcriptomic response to aluminum stress between resistant and sensitive genotypes to identify genes potentially involved in aluminum tolerance. We developed the scoring that takes into account gene expression changes within resistant and sensitive groups of genotypes, their consistency, and differences between groups. This allowed us to find out transcripts with diverse expression alterations between groups of resistant and sensitive to aluminum flax genotypes but similar alterations within resistant or sensitive groups. Results of the analysis are presented in S8 Table. Transcripts were sorted by decreasing delta-score that reflects differences in Al^3+^-induced transcriptomic response between resistant and sensitive groups of genotypes. Within upregulated transcripts in resistant genotypes compared to sensitive ones, transcripts encoding Agamous-like MADS-box protein AGL62, polygalacturonase, NAC domain-containing protein 100, protein OSB1, GDSL esterase/lipase, beta-glucosidase 11, (+)-neomenthol dehydrogenase, osmotin-like protein OSML13, and 1-aminocyclopropane-1-carboxylate oxidase homolog 1 were in the top.

First of all, our attention was attracted by genes that were upregulated only in resistant cultivars under Al^3+^ exposure. In the top of S8 Table, two TFs are located: Agamous-like MADS-box protein AGL62 and NAC domain-containing protein 100. MADS-box genes control numerous aspects of plant development and are involved in stress response [52–55]. NAC TFs also participate in stress response and their overexpression in transgenic plants enhances tolerance to abiotic stresses and promotes development of lateral roots [56–61]. Role of NAC in response to aluminum was revealed in maize and rice; these TFs were involved in phytohormone signaling and growth regulation [62–64]. In our study, expression of transcript TR35721∣c0_g1, which encodes Agamous-like MADS-box protein AGL62, was induced by Al only in resistant flax genotypes (the binary logarithm of fold change, log⁡*FC*, was equal to 2.3 and 3.0 in Hermes and TMP1919, respectively). The same was true for transcript TR25300∣c0_g1 encoding NAC domain-containing protein 100: significant upregulation was revealed under aluminum stress in resistant cultivars (log⁡*FC* was equal to 1.0 and 1.1 in Hermes and TMP1919, respectively), while some decrease of the expression—in sensitive ones. Thus, overexpression of genes encoding NAC domain-containing protein 100 and Agamous-like MADS-box protein AGL62 only in resistant flax genotypes under Al^3+^ exposure indicates that these TFs could contribute to flax tolerance to aluminum. We also analyzed mRNA level alterations of other TFs, whose role in response to Al is known. The role of WRKY TFs in Al tolerance in plants was shown to be ambiguous: in* Arabidopsis*, WRKY46 was identified as a negative regulator of ALMT1 and mutations in WRKY46 lead to increased malate secretion and higher Al resistance [65]; on the contrary, WRKY22 promoted aluminum tolerance in rice through activation of FRDL4 (Ferric reductase defective 4) and increased citrate secretion [66]. In flax, we revealed slight upregulation of a number of WRKY-encoding genes but did not observe differences between resistant and sensitive cultivars. For genes encoding sensitive to proton rhizotoxicity 1 (STOP1) and Abscisic acid stress ripening (ASR), which are also known TFs associated with aluminum response [17, 20, 67–69], no significant expression alterations were revealed by us in flax under aluminum stress. Thus, it can be suggested that Agamous-like MADS-box protein AGL62 and NAC domain-containing protein 100 are the leading TFs involved in Al tolerance in flax* via* regulation of plant growth and development.

Besides TFs, we observed more pronounced upregulation of genes encoding polygalacturonase, cellulose synthase, pectinesterase, beta-glucosidase, and GDSL esterase in resistant to aluminum flax cultivars compared to sensitive ones. These enzymes are involved in cell wall metabolism, which plays important role in plant response to heavy metals and other abiotic stresses [70]. Modification of cell wall and changes in binding properties of the apoplast are known to contribute to Al tolerance in plants [70, 71]. In flax, upregulation of genes encoding cell wall-related proteins was observed in resistant genotypes; therefore, cell wall modification could be one of the mechanisms of flax tolerance to Al ions.

In the top of the list of transcripts that were differentially expressed between resistant and sensitive genotypes (S8 Table), we also observed peroxidase and ABC transporter-encoding genes, whose role in plant tolerance to aluminum stress is known [20]. Peroxidases are involved in detoxification of ROS and their overexpression in some plant species is associated with Al tolerance [20]. Moreover, overexpression of* Arabidopsis* peroxidase in tobacco plants improved their tolerance to aluminum [72]. At the same time, peroxidase expression was decreased under aluminum treatment in* Camellia sinensis* [73]. In wheat, peroxidase expression was induced by Al stress; however, their activity was lower in resistant genotypes compared to sensitive ones [74, 75], while in chickpea, peroxidase activity was almost similar in tolerant and sensitive genotypes [76]. In our study on flax, for TR33816∣c0_g1 transcript encoding peroxidase 5, a significant expression decrease was revealed in resistant genotypes (log⁡*FC* was equal to -1.7 for both Hermes and TMP1919), while slight decrease or retention was identified in sensitive ones (log⁡*FC* = −0.2 for Lira and log⁡*FC* = −0.5 for Orshanskiy). The role of ABC transporters in plant response to aluminum stress is well characterized [8, 77–81]. In flax, ABC transporter-encoding transcript TR4576∣c0_g2 had diverse expression changes between resistant and sensitive to aluminum genotypes: strong expression decrease was revealed in resistant cultivars (log⁡*FC* was equal to -1.7 and -1.2 for Hermes and TMP1919, respectively) and slight expression decrease was observed in sensitive ones (log⁡*FC* = −0.3 for Lira and log⁡*FC* = −0.4 for Orshanskiy). For ABC transporter, we did not find associations of flax tolerance to Al with high expression levels of transcripts both under stress and control conditions. Considering the fact that expression of aluminum tolerance genes is usually higher in resistant genotypes and often increased by Al treatment [8], we suggested that peroxidase- and ABC transporter-encoding genes are not the aluminum tolerance gene in flax.

The scoring for identification of differentially expressed genes in resistant genotypes compared to sensitive ones under Al^3+^ exposure was shown to be the promising tool for revelation flax genes that are involved in tolerance to aluminum. Obtained results allowed us to suggest that some genes (including STOP1-, ASR-, peroxidase-, and ABC transporter-encoding ones), whose role in aluminum response was revealed for several plant species, do not play the key role in flax tolerance to the stress. At the same time, MADS-box and NAC TFs, which regulate plant growth and development, and the enzymes that are involved in cell wall modifications are likely important for flax tolerance to aluminum.

### 3.3. GO Analysis and Gene Expression Profiles

**Figure 2:**
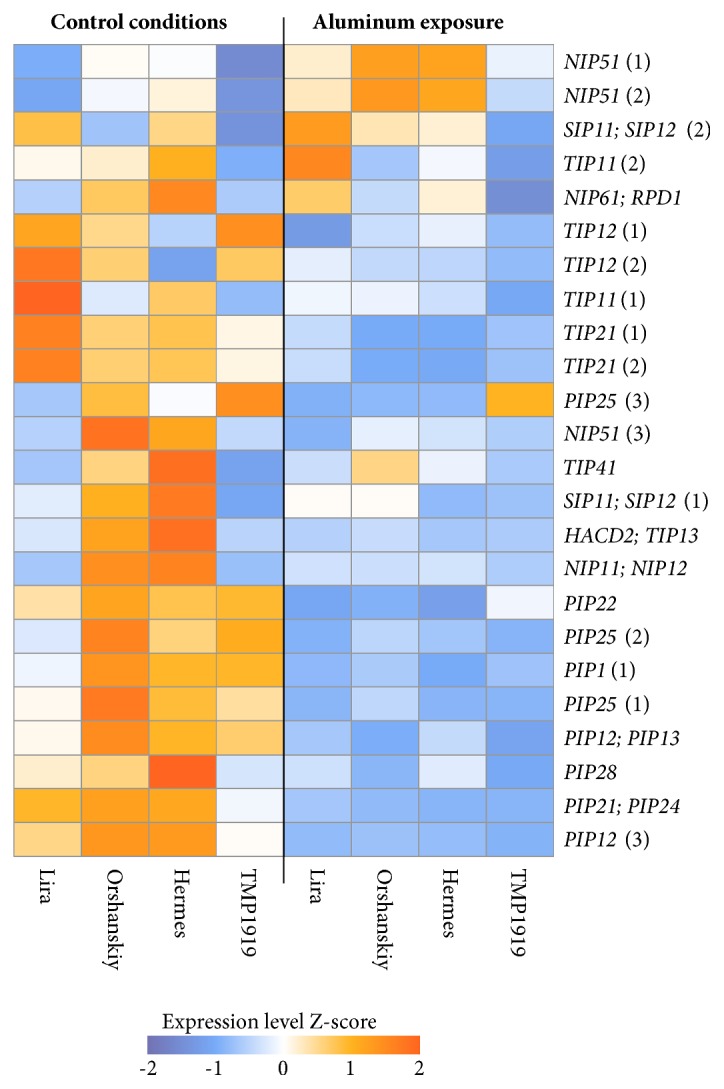
**Expression patterns for genes participating in water transmembrane transporter activity (GO 0005372) in flax cultivars Lira, Orshanskiy, Hermes, and TMP1919 under control conditions and aluminum exposure.** The heatmap represents Z-scores of normalized read counts per million (CPM) for each gene: from blue (low expression level) to orange (high expression level). Row names show top BLAST hits of the assembled gene transcripts.

For deeper understanding of the mechanisms of flax response to aluminum, we performed GO enrichment analysis to identify the processes in which genes with significant expression alterations under Al^3+^ exposure are involved. Overrepresented GO terms were assessed for top lists (tops) of up- and downregulated genes in flax plants under aluminum exposure. Tops of 50, 100, 300, and more than 300 differentially expressed genes (maximum number of genes was limited by the statistical significance of expression alterations corresponding to* p-value* < 0.05 in S1, S2, and S3 Tables) were used in the analysis. For the pool of all studied cultivars, overrepresented GO terms for the tops of 50 and 100 upregulated genes were related to circadian rhythm, multicellular organismal movement, and potassium: sodium symporter activity, while for the top of 300 upregulated genes, transferase activity had also appeared (S9 Table). For all the tops of downregulated genes for the pool of all studied genotypes, GO terms related to channel activity and transport were the most abundant. Heatmap for “water transmembrane transporter activity” GO term is presented in Figure 2 as an example. In the pool of sensitive to aluminum flax genotypes, we identified less number of significantly overrepresented GO terms compared to the pool of resistant ones for the tops of upregulated genes (S10 and S11 Tables). These GO terms were associated with apoplast, rhythmic processes, peptidase and oxidoreductase activities. In resistant cultivars, overrepresented GO terms included oxidoreductase activity and rhythmic processes too, but unlike sensitive genotypes, GO terms related to glutathione metabolism, glucosyltransferase activity, and response to stimulus were also overrepresented.

**Figure 3:**
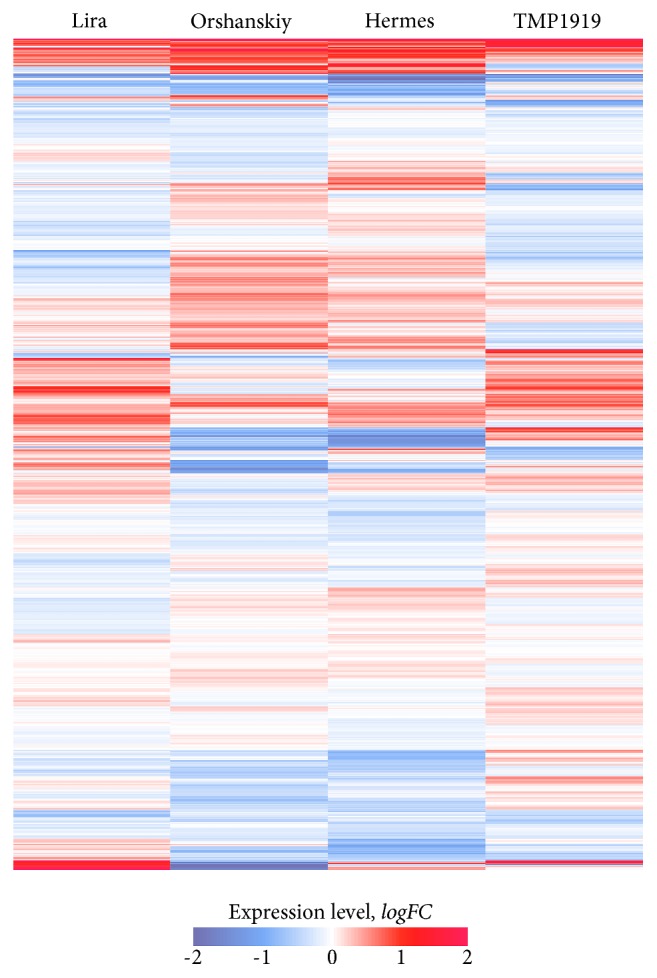
**Expression alterations in flax plants under aluminum exposure in Lira, Orshanskiy, Hermes, and TMP1919 cultivars for genes involved in oxidoreductase activity (GO 0016491).** Each heatmap row corresponds to one gene (total 21 000 genes). Color scale represents the binary logarithm of expression level fold change (aluminum exposure/control conditions) from -2 (i.e., 4-fold downregulation, blue) to +2 (4-fold upregulation, red).

We also performed GO analysis for individual cultivars to evaluate the similarity in response to aluminum of genotypes of the same tolerance group (S12, S13, S14, and S15 Tables). For the top of 50 upregulated genes, glutathione transferase activity term was overrepresented in both resistant cultivars (Hermes and TMP1919). However, for TMP1919, unlike Hermes, oxidoreductase activity term was also overrepresented and, in this, it was similar to sensitive cultivar Orshanskiy. When the top of 300 upregulated genes was used for analysis, much more GO terms were overrepresented. Glutathione transferase activity was in the top of the list not only in resistant genotypes but also in sensitive cultivar Orshanskiy, while oxidoreductase activity term was overrepresented in all studied genotypes. Gene expression profiles of particular GO terms had no relation to the degree of tolerance to aluminum. For example, expression alterations of genes related to oxidoreductase activity were more similar for pairs of cultivars TMP1919/Lira and Hermes/Orshanskiy (Figure 3).

GO enrichment analysis allowed us to reveal biological processes that are the most affected by aluminum stress in flax. Summarizing the results of this analysis, it could be concluded that Al^3+^ exposure had negative effects on transmembrane transport in flax. It is not surprising since aluminum disturbs ion transport in plants [19]. It is known that oxidase overexpression confers aluminum tolerance of plants [82, 83] and genes related to oxidoreductase activity GO term were upregulated in response to aluminum in flax. For glutathione transferase activity GO term, alterations in gene expression were more pronounced in resistant cultivars. Their role in Al response was discussed in our previous study [42].

To reveal coexpression gene networks in flax under aluminum stress, weighted gene coexpression network analysis (WGCNA) was performed for 8986 genes with CPM more than 20 in 6 or more samples. We identified 23 clusters (modules) of coexpressed genes based on similarity in gene expression (S16 Figure). Modules included from 46 to 1831 genes: module 1 was formed by 1831 genes enriched for RNA metabolic process and proton antiporter activity; module 2—organelle membrane and oxidation-reduction process; 3—ribosome and macromolecule biosynthetic process; 6—transporter activity and rhythmic process; 7—regulation of cellular process and macromolecule biosynthesis; 8—phosphorus metabolic process, ion binding, and oxidation-reduction process; 9—nucleolus, ribosome, and RNA metabolic process; 13—mitochondrial part; 14—ion transport; 16—lipid metabolic processes. Genes from the same module are probably coregulated, and the processes, in which they are involved, may be related to each other. For other modules, we did not reveal significant enrichment in genes of particular GO terms.

## 4. Conclusions

We analyzed transcriptomes of flax cultivars with diverse tolerance to aluminum under control conditions and Al stress and identified genes that were significantly up- or downregulated. A number of genes whose expression alterations differed between resistant and sensitive cultivars were revealed, including those encoding MADS-box and NAC TFs and cell wall biogenesis related enzymes, suggesting them to be involved in aluminum tolerance in flax. These results indicate that, for flax, the internal tolerance to aluminum* via* reduction of Al ions toxicity is inherited. GO enrichment analysis led to the identification of processes that are affected by aluminum in both resistant and sensitive flax genotypes. The genotype effect on the response to Al^3+^ exposure was strong enough; although resistant and sensitive cultivars had diverse gene expression changes under the stress, significant impact of a particular genotype on stress response was also observed. Understanding the mechanisms of flax response to Al and identification of tolerance genes is the basis for the development of improved cultivars which will retain high productivity on acid soils that is more preferable strategy than liming the soils.

## Figures and Tables

**Table 1:** Primers and probes for qPCR.

**Primer name**	**Primer sequence**	**Probe number from Roche Universal ProbeLibrary**
LHY-F	CAGGAATCGAAGTTGGGAGA	12
LHY-R	CGCTGCTTCAAATCCTCTCTAA	

LUX-F	GGGGAGTGGATGCAAAGAG	69
LUX-R	CGACTTTACCTCAAGAGGATGC	

JMJ-F	GAACCATCTTTGCCCTGAATC	48
JMJ-R	AGGAGTGGAAGCAAGAGCTG	

HMGB-F	GCTTTCTCTGCACTAGACAAAGATT	7
HMGB-R	AACAAAGCTGTCTCCGCTGT	

COL-F	CGTATGAATTCAATGCAGCAG	66
COL-R	CAAACAGCTGGTTCGGTTTTA	

NRT-F	ATTTTGAACTGGCAGGTCTTG	69
NRT-R	CGGTGAGCCAGAAGGACA	

APRR-F	CGCTGAAGTTGATCTTCCAAT	89
APRR-R	GCAAATCCTTATGCCGAGTT	

ETIF3E-F	TTACTGTCGCATCCATCAGC*∗*	53
ETIF3E-R	GGAGTTGCGGATGAGGTTTA*∗*	

*Note.* Primers were designed for genes encoding protein LHY (LHY, TR32133∣c1_g1), TF LUX (LUX, TR44570∣c0_g2), putative lysine-specific demethylase JMJ30 (JMJ, TR19190∣c0_g1), high mobility group B protein 1 (HMGB, TR12175∣c1_g1), zinc finger protein CONSTANS-LIKE 10 (COL, TR53996∣c0_g1), high-affinity nitrate transporter 3.1 (NRT, TR26619∣c0_g1), and two-component response regulator-like APRR1 (APRR, TR32104∣c0_g2). *∗*: Primer sequences for *ETIF3E* are from Huis et al. article [47].

**Table 2:** Phenotype changes in flax plants under aluminum treatment at low pH.

Cultivar	Condition	Plant height, cm	Decline, %	Fiber mass, mg	Decline, %	Number of seed pods	Decline, %
Hermes	Stress	60.6±0.9	85.9	115.6±5.6	85.3	2.9±0.4	96.6
	Control	70.5±0.9		135.6±8.2		2.8±0.4	

ТМР1919	Stress	69.4±1.6	94.2	93.7±3.8	86.0	3.5±0.3	81.4
	Control	73.7±1.7		108.9±5.4		4.3±0.5	

Lira	Stress	56.2±1.2	75.3	54.4±5.8	41.3	2.5±0.3	56.8
	Control	74.6±1.5		131.8±7.4		4.4±0.3	

Orshanskiy	Stress	61.2±1.4	86.2	52.8±4.4	63.1	3.2±0.3	59.3
	Control	71.0±0.6		83.7±8.3		5.4±0.4	

*Note.* Stress: aluminum exposure at pH 4.5.

## Data Availability

Sequencing data used to support the findings of this study have been deposited in Sequence Read Archive–SRP089959. Transcript sequences and annotations could be downloaded from the following link: http://194.226.21.15/Flax.Aluminium/. Gene expression data used to support the findings of this study are included in the Supplementary Files.

## Supplementary Materials

Supplementary Materials S1 Table. Gene expression alterations in all analyzed flax genotypes under aluminum stress. S2 Table. Gene expression alterations in resistant flax genotypes under aluminum stress. S3 Table. Gene expression alterations in sensitive flax genotypes under aluminum stress. S4 Table. Gene expression alterations in resistant flax cultivar Hermes under aluminum stress. S5 Table. Gene expression alterations in resistant flax cultivar TMP1919 under aluminum stress. S6 Table. Gene expression alterations in sensitive flax cultivar Lira under aluminum stress. S7 Table. Gene expression alterations in sensitive flax cultivar Orshanskiy under aluminum stress. S8 Table. Diverse expression alterations between groups of resistant and sensitive to aluminum flax genotypes. S9 Table. GO enrichment analysis for tops of up- and downregulated genes in the pool of all studied flax cultivars after aluminum exposure. S10 Table. GO enrichment analysis for tops of up- and downregulated genes in the pool of resistant flax cultivars after aluminum exposure. S11 Table. GO enrichment analysis for tops of up- and downregulated genes in the pool of sensitive flax cultivars after aluminum exposure. S12 Table. GO enrichment analysis for tops of up- and downregulated genes in resistant flax cultivar Hermes after aluminum exposure. S13 Table. GO enrichment analysis for tops of up- and downregulated genes in resistant flax cultivar TMP1919 after aluminum exposure. S14 Table. GO enrichment analysis for tops of up- and downregulated genes in sensitive flax cultivar Lira after aluminum exposure. S15 Table. GO enrichment analysis for tops of up- and downregulated genes in sensitive flax cultivar Orshanskiy after aluminum exposure. S16 Figure. Hierarchical cluster dendrogram showing coexpression modules identified using weighted gene coexpression network analysis (WGCNA) for flax under aluminum stress.** Symbols in Supplementary Tables:** Gene-gene identifier (generated by Trinity). Transcript lengths: comma-separated lengths of all predicted transcripts. Blast symbol: gene symbol of top BLAST hit(s). Blast name: gene name of top BLAST hit(s). Blast species: organism to which top BLAST hit(s) belongs. KEGG Orthology: KEGG BRITE gene/protein identifier (pan-organismal) of the top BLAST hit. KEGG A.Thaliana: KEGG gene/protein identifier (*Arabidopsis*  *thaliana*) of the top BLAST hit. Gene Ontology: Gene Ontology annotation of the top BLAST hit. logFC: binary logarithm of gene expression level fold change between intact and Al-treated plants (an average across all four cultivars). logCPM: binary logarithm of read counts per million (average across all the samples). LR: likelihood ratio from edgeR's likelihood ratio test (LRT). PValue: LRT p-value. FDR: adjusted LRT p-value. Hermes-C: CPM (Hermes, control). Hermes-Al4: CPM (Hermes, Al-treated for 4 hours). TMP-C: CPM (TMP1919, control). TMP-Al4: CPM (TMP1919, Al-treated for 4 hours). Lira-C: read counts per million (CPM) per gene (Lira, control). Lira-Al4: CPM (Lira, Al-treated for 4 hours). Orshanskiy-C: CPM (Orshanskiy, control). Orshanskiy-Al4: CPM (Orshanskiy, Al-treated for 4 hours).
