# Supplementary figures and images for: Aluminum Responsive Genes in Flax (Linum usitatissimum L.)
Source: Biomed Res Int. 2019 Feb 28;2019:5023125. doi: 10.1155/2019/5023125 (PMC6421055; doi:10.1155/2019/5023125)

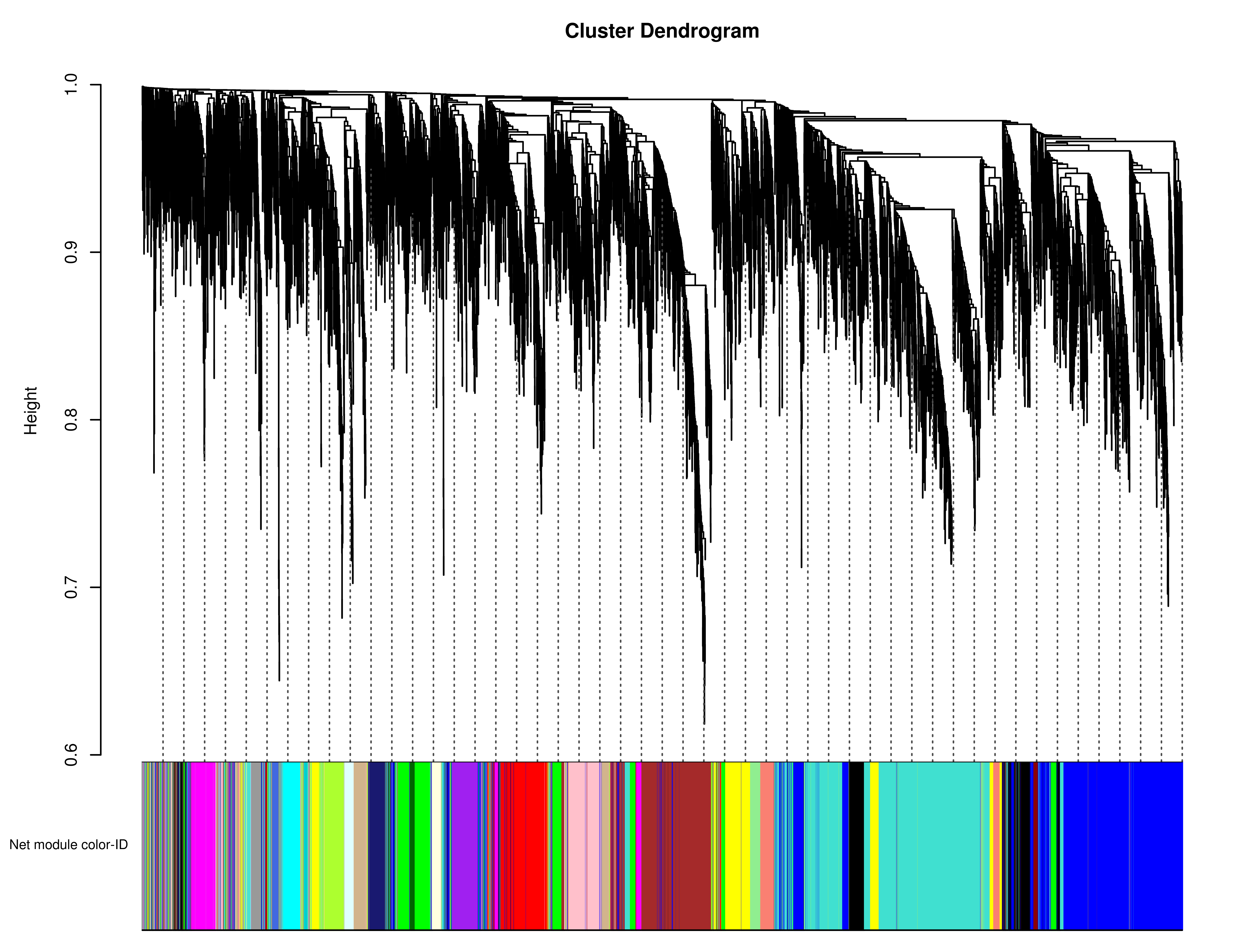

Supplement: Supplementary Materials — S1 Table. Gene expression alterations in all analyzed flax genotypes under aluminum stress. S2 Table. Gene expression alterations in resistant flax genotypes under aluminum stress. S3 Table. Gene expression alterations in sensitive flax genotypes under aluminum stress. S4 Table. Gene expression alterations in resistant flax cultivar Hermes under aluminum stress. S5 Table. Gene expression alterations in resistant flax cultivar TMP1919 under aluminum stress. S6 Table. Gene expression alterations in sensitive flax cultivar Lira under aluminum stress. S7 Table. Gene expression alterations in sensitive flax cultivar Orshanskiy under aluminum stress. S8 Table. Diverse expression alterations between groups of resistant and sensitive to aluminum flax genotypes. S9 Table. GO enrichment analysis for tops of up- and downregulated genes in the pool of all studied flax cultivars after aluminum exposure. S10 Table. GO enrichment analysis for tops of up- and downregulated genes in the pool of resistant flax cultivars after aluminum exposure. S11 Table. GO enrichment analysis for tops of up- and downregulated genes in the pool of sensitive flax cultivars after aluminum exposure. S12 Table. GO enrichment analysis for tops of up- and downregulated genes in resistant flax cultivar Hermes after aluminum exposure. S13 Table. GO enrichment analysis for tops of up- and downregulated genes in resistant flax cultivar TMP1919 after aluminum exposure. S14 Table. GO enrichment analysis for tops of up- and downregulated genes in sensitive flax cultivar Lira after aluminum exposure. S15 Table. GO enrichment analysis for tops of up- and downregulated genes in sensitive flax cultivar Orshanskiy after aluminum exposure. S16 Figure. Hierarchical cluster dendrogram showing coexpression modules identified using weighted gene coexpression network analysis (WGCNA) for flax under aluminum stress. Symbols in Supplementary Tables: Gene-gene identifier (generated by Trinity). Transcript lengths: comma-separ [file 5023125.f1.zip › 5023125.f1/S16 Figure.png]
